# Moderators of Dual Task Gait Effects in Mild Cognitive Impairment and Dementia

**DOI:** 10.1093/geroni/igab046.3184

**Published:** 2021-12-17

**Authors:** Edward Ofori, Dara James, Olivia Kaczmarek, Mark Gudesblatt

**Affiliations:** 1 ASU, Arizona State University, Arizona, United States; 2 Arizona State University, Phoenix, Arizona, United States; 3 SouthShore Neurologic, Southshore Neurologic, New York, United States; 4 South Shore Neurologic, Islip, New York, United States

## Abstract

Spatiotemporal gait parameters may provide indication about the cognitive status of individuals. Dysfunction in specific gait features has been associated with increased risk of cognitive decline. Here we use spatiotemporal gait patterns to determine whether specific cognitive domain scores moderate the effects during dual-tasking on individuals with mild cognitive impairment (MCI) and dementia. Participants (n=46; mean age: 77.0±8.9 years) with a diagnosis of cognitive impairment (n=16), or dementia (n=30) were included. They performed validated computerized cognitive assessment battery (CAB, NeuroTrax BrainCare) to obtain cognitive domain measures of executive function (EF), attention, memory, visual-spatial processing (VSP), information processing speed (IPS), and a global cognitive score (GCS) measure. Using the Zeno Walkway Gait Analysis System (Protokinetics), measures of velocity, stride width (SW), stride time (ST), stride length, cadence, double support (DS), and gait variability were obtained for both single-task and DT gait. Data analysis was conducted using SPSS 26 and PROCESS 3.5. As expected, the dementia group had lower cognitive domain scores and slower walking speed than MCI group. Results also indicated that visual-spatial processing skills was the only cognitive domain that did have a moderation effect on gait velocity (F=4.2, p<0.05, R-square change 10%). Our results indicate that differences between walking speed in MCI and dementia groups are moderated by visual spatial skills. Improvement in visual spatial skills could improve the dual task effects of individual gait measures.

